# Rethinking the biology of metastatic melanoma: a holistic approach

**DOI:** 10.1007/s10555-021-09960-8

**Published:** 2021-04-19

**Authors:** Hendrik HLD Vandyck, Lisa M Hillen, Francesca M Bosisio, Joost van den Oord, Axel zur Hausen, Véronique Winnepenninckx

**Affiliations:** 1 Department of Pathology, GROW-School for Oncology & Developmental Biology, Maastricht University Medical Center, MUMC+, PO Box 5800, 6202 AZ Maastricht, The Netherlands; 2 Laboratory of Translational Cell and Tissue Research (TCTR), Department of Pathology, KU Leuven and UZ Leuven, Leuven, Belgium

**Keywords:** Melanoma, Metastasis, Epithelial-mesenchymal transition, Phenotype switching, Dormancy, Warburg effect

## Abstract

Over the past decades, melanoma-related mortality has remained nearly stable. The main reason is treatment failure of metastatic disease and the inherently linked knowledge gap regarding metastasis formation. In order to elicit invasion, melanoma cells manipulate the tumor microenvironment, gain motility, and adhere to the extracellular matrix and cancer-associated fibroblasts. Melanoma cells thereby express different cell adhesion molecules like laminins, integrins, N-cadherin, and others. Epithelial-mesenchymal transition (EMT) is physiological during embryologic development, but reactivated during malignancy. Despite not being truly epithelial, neural crest-derived malignancies like melanoma share similar biological programs that enable tumorigenesis, invasion, and metastasis. This complex phenomenon is termed phenotype switching and is intertwined with oncometabolism as well as dormancy escape. Additionally, it has been shown that primary melanoma shed exosomes that create a favorable premetastatic niche in the microenvironment of secondary organs and lymph nodes. Although the growing body of literature describes the aforementioned concepts separately, an integrative holistic approach is missing. Using melanoma as a tumor model, this review will shed light on these complex biological principles in an attempt to clarify the mechanistic metastatic pathways that dictate tumor and patient fate.

## **Introduction**

Cutaneous malignant melanoma is a relatively rare type of skin cancer but accounts for 73% of skin cancer-related deaths worldwide with an incidence that continues to rise [1]. Metastatic melanoma mostly has a fatal course as the 5-year overall survival rate drops to 23% in stage IV patients [1]. Despite recent therapeutic advances provided by immunotherapy and targeted drugs, therapy resistance and disease recurrence usually are reality [2]. Drug resistance in minimal residual disease (MRD) is not only caused by “Darwinian selection” of specific genetic mutations but also by adaptive non-mutational “Lamarckian induction” [3–5]. Successful metastasis is accomplished by the five key steps of the metastatic cascade: invasion, intravasation, circulation, extravasation, and colonization at secondary tumor sites [6].

Epithelial-mesenchymal transition (EMT) is a cellular program essential during embryogenesis that is revived during malignancy [7, 8]. In EMT, epithelial markers are downregulated while mesenchymal markers are upregulated [9]. Moreover cell-cell and cell-matrix interplay is remodeled allowing subsequent motility and migration of cancer cells. This is accompanied by changes in adhesion molecules on cancer cells and the adjacent cancer-associated fibroblasts (CAFs) [10, 11]. An overwhelming body of literature focuses on EMT in carcinomas; yet, similar mechanisms drive melanomagenesis in which the term phenotype switching is preferred [7, 12]. The resulting melanoma cell plasticity leads to an increased sensitivity and adaptation to microenvironmental changes. Closely related is metabolic flexibility that depends on nutrient availability, hypoxia and reactive oxygen species (ROS) [13–15]. Here, the right context-dependent metabolic “sweet spot” is achieved by utilizing the amount of glucose that is present to meet the minimal viable needs yet maximal anabolic potential. In addition to cooperation with CAFs, metabolic symbiosis is amplified by nutrient trade-off and interphenotypic communication between melanoma subsets [16–20]. The established intercellular “social” interaction results in tumors of greater fitness [16, 17].

Nevertheless, macrometastatic outgrowth remains a highly inefficient process, in part because of the plethora of environmental noxae that kill unadapted tumor cells after intravasation [21]. If disseminated melanoma cells survive, they often undergo a dormant state that is featured by a high survival phenotype at the expense of proliferation [22, 23]. This dormant state as well as its reversion (i.e., dormancy escape) is inherently linked with a metabolic and phenotypical switch. Nonetheless, dormancy escape and macrometastatic outgrowth is only possible if adaptation to the new microenvironment is successful. Stated differently, not only epigenetic reprogramming in a background of preexisting genetic alterations must suffice, but also a favorable distant microenvironment that defines tumor-specific organotropism must be present. Interestingly, primary melanomas shed exosomes that create “fertile soils”, or premetastatic niches at secondary sites that are receptive and supportive for tumor colonization [24, 25]. Fundamental knowledge in depth about the mechanistics of metastasis formation is indispensable before considering new therapeutic approaches.

In this review, we will discuss the metastatic melanoma-driving biological principles of (i) EMT-related phenotype switching, (ii) the essential roles of the tumor microenvironment (TME) and melanoma cell adhesion, (iii) metabolic reprogramming and synergy with the stroma, (iv) melanoma cell dormancy, and (v) melanoma cell exosomes. Moreover, we will elaborate on the unmistakable reciprocity of these concepts as well as the recent advances of mechanistic insights in melanomagenesis and metastasis formation. Finally, we will provide a brief outlook on pending future research questions in the field of metastatic melanoma. We thereby aim to help uncover the missing links of the confounding mechanisms of metastasis that remain an ongoing matter of debate.

## EMT and phenotype switching

### General principles of EMT

**Fig. 1:**
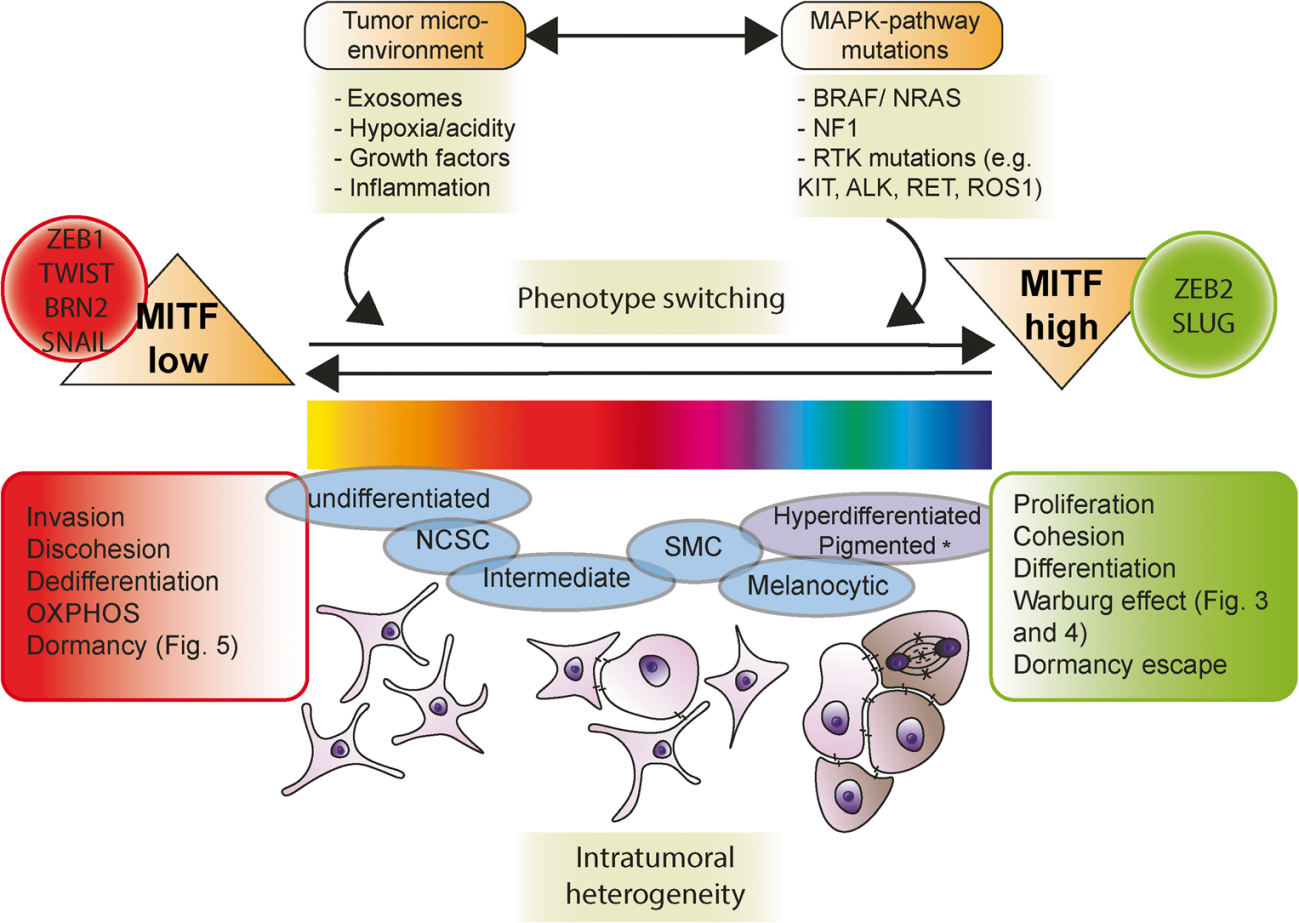
Phenotype switching in melanoma. Proposed model of phenotype switching in melanoma with integrative reciprocity of dormancy, metabolic reprogramming, and role of the TME. Phenotype switching is orchestrated by EMT-TFs and involves MITF^low^ and MITF^high^ interchangeable states that provide context-dependent malignant potential. Note that multiple intermediate states exist and that phenotype switching in melanoma is not a binary process, just as EMT/MET is only partial in carcinogenesis. The recently discovered distinct transcriptional melanoma cell states include: undifferentiated, NCSC, intermediate, SMC, melanocyte-like and hyperdifferentiated/pigmented state. The different melanoma cell states predominantly are the result of epigenetic induction that contribute to plasticity, reversibility and therapy resistance. Adopted from [5, 7, 27]. Abbreviations: EMT, epithelial-mesenchymal transition; MET, mesenchymal epithelial transition; MITF, microphtalmia transcription factor; NCSC, neural crest stem cell; NF-1, neurofibromin 1; RTK, receptor tyrosine kinase; SMC, starved melanoma cell; TF, transcription factor

EMT is a cellular program essential during embryogenesis, fibrosis, wound healing and malignancy [8]. In EMT epithelial markers like cytokeratins, E-cadherin and occludins among others are downregulated while mesenchymal markers like vimentin and alpha-smooth muscle actin (αSMA) are upregulated [9]. These underlying molecular changes are accompanied by phenotypic alterations including the morphologic change from an epithelioid towards a mesenchymal/spindle cell shape. This process is orchestrated by EMT-inducing transcription factors (EMT-TFs) such as TWIST1 and TWIST2, ZEB1 and ZEB2, and SNAIL and SLUG [9, 26] (Fig. 1). EMT-TFs act in a highly pleiotropic, cell context-dependent manner in various combinations to express and suppress a plethora of genes [8, 26, 28]. Rather than being a binary process, the progressive loss of epithelioid and gain of mesenchymal features is mainly partial in cancer [29]. The following more mesenchymal state is reverted *via* mesenchymal-epithelial transition (MET) that precedes the formation of the metastasis [30]. Just as in embryological development and its reversible physiological state of EMT-MET, the underlying mechanism of metastatic dissemination is believed not to be predominantly driven by sequential genetic mutations [6, 30]. Instead, plasticity and reversibility are needed for redifferentiation towards the MET phenotype. This may explain why EMT-TFs are only seldom mutated despite their overt oncogenic potential [30–32]. Rather they are fine-tuned by transcriptional, translational, posttranslational and other epigenetic mechanisms [32, 33]. Double strand DNA breaks are one of the most damaging and apoptosis inducing subcellular events [34]. EMT-TFs can induce repair of double strand DNA breaks, chromosomal instability, apoptosis resistance and senescence escape [26]. Indeed, EMT-TFs have additional oncologic effects beyond merely the transition towards a mesenchymal phenotype and back. For instance, EMT-TFs also contribute to cancer progression through immune evasion [35].

### Melanomagenesis and metastasis are driven by phenotype switching

Although a generally accepted term, EMT describes the abovementioned processes in carcinomas, whereas expanding data suggest explicit roles of EMT-TFs also in non-epithelial malignancies (for overview, see Table 1). Thus, phenotype switching is a more general but possibly more appropriate term when considering the EMT-like processes in melanoma. Transdifferentiation on the other hand is better defined as exiting the (melanocytic) lineage to a different cell lineage like endothelial cells or CAFs [7, 63]. Melanoma cell plasticity encompasses both phenotype switching and transdifferentiation. Although EMT does not exist in melanocytes as melanocytes are not true epithelial cells, a complete EMT program does occur in the formation, delamination and migration of (neuroepithelial) neural crest (NC) cells during embryologic development [64]. Instead, melanomas undergo phenotype switching which enables their striking invasive and disseminating properties [7, 65]. NC cells are multipotent transient cells that migrate and seed to different tissues to differentiate into specific cell lineages like melanocytes [7]. In case of NC derivatives, ZEB2 has been proven to be indispensable for terminal differentiation *in vivo* for melanocytes, oligodendrocytes and Schwann cells by upregulation of the microphthalmia-associated transcription factor (MITF) [66, 67]. Once arrived in the epidermis, melanocytes express adhesion molecules like E-cadherin just as their neighboring basal keratinocytes. Similar as in NC-derived melanoblasts, the cadherin-switch (from E-cadherin to N-cadherin) takes place in an important subset of melanomas and is induced by ZEB1, TWIST and SNAIL [7, 68, 69]. Desmoplastic melanomas typically are very invasive, poorly demarcated melanomas with an obvious spindle cell morphology [70]. This is in line with EMT and the more often observed cadherin-switch in desmoplastic melanoma [71]. Moreover, they often immunohistochemically lack the pigmentation markers MITF, Melan-A, Human Melanoma Black 45 (HMB45) and gain αSMA [70, 71].

**Table 1:** Examples of epithelial and non-epithelial derived malignancies in which EMT has essential roles in tumorigenesis, progression, metastasis and therapy resistance

**Tumor type examples**	**Observations that link EMT to cancer (epithelial)**	**Reference**
Breast	SNAIL expression is observed in invasive ductal carcinomas and correlates with lymph node metastasis	Moody et al., 2005 [36]
Breast	TWIST1 promotes metastasis of mouse mammary carcinomas	Yang et al., 2004 [37]
Breast	HER2-induced mammary tumors spontaneously express SNAIL and express features of EMT	Blanco et al., 2002 [38]
Breast	SNAIL expression is observed during carcinoma progression in an autochthonous model of breast cancer	Ye et al., 2015 [39]
Pancreatic	Invasive carcinoma cells exhibit features of EMT in an autochthonous mouse model of pancreatic cancer	Rhim et al., 2012 [40]
Pancreatic	ZEB1 strongly impacts tumor progression, invasion and metastasis by inducing stemness in pancreatic cancer	Krebs et al., 2017 [41]; Lemma et al., 2013 [42]
Lung	The expression of EMT markers is tightly associated with disease progression in NSCLC	Prudkin et al., 2009 [43]
Lung	EMT markers are expressed at the peripheral leading edge of NSCLC, and marker presence is correlated with tumor progression	Mahmood et al., 2017 [44]
Colorectal	SLUG expression is correlated with tumor progression and is a marker for poor prognosis in patients	Shioiri et al., 2006 [45]
Colorectal	ZEB2 is expressed at the invasive front, which correlates with tumor progression and is a prognostic marker for colorectal cancer	Kahlert et al., 2011 [46]
Colorectal	N-cadherin drives malignant progression of colorectal cancer	Yan et al., 2015 [47]
Hepatocellular	Overexpression of TWIST induces EMT and promotes invasion and metastasis of hepatocellular carcinomas	Lee et al., 2006 [48]
Hepatocellular	SNAIL induces EMT and promotes metastasis and tumor-initiating properties in hepatocellular carcinomas	Zhou et al., 2014 [49]
Bladder	EMT markers are associated with tumors of high grade and stage	Baumgart et al., 2007 [50]
Bladder	SNAIL-induced EMT promotes metastasis in a xenograft model of bladder cancer	Roth et al., 2017 [51]
Bladder	E-cadherin is negatively correlated with tumor grade and stage, while expression of SOX2 and NANOG positively correlates with those clinicopathological parameters.	Migita et al., 2017 [52]
Prostate	A switch from E-cadherin to N-cadherin shows significant associations with prostate cancer progression in patients	Gravdal et al. 2007 [53]
Prostate	TWIST expression is higher in tumor tissue than in benign prostate hyperplasia and correlates with Gleason grade >7; TWIST expression is also increased in bone and lymph node metastases	Kwok et al., 2005 [54]
**Tumor type examples**	**Observations that link EMT to cancer (non-epithelial)**	**Reference**
Leukemia/lymphoma	ZEB2-overexpression in immature as well as in more differentiated T-cell precursors drives malignant T-cell development	Goossens et al., 2019 [55]
Leukemia/lymphoma	ZEB1 is associated with adverse clinical presentation and clinical outcome, whereas cytoplasmatic SLUG expression is linked to a favorable prognosis in DLBCL	Lemma et al., 2013 [42]
Multiple myeloma (MM)	TWIST1 expression is elevated in skeletal extramedullary disease of patients with MM and correlates with a lower rate of progression-free survival	Yang et al., 2016 [56]
MM	Hypoxia drives mesenchymal(-like) transition in MM cells by a decrease in E-cadherin levels and increasement in EMT-inducing proteins such as SNAIL and TGF-β	Azab et al., 2012 [57]
Glioblastoma multiforme (GBM)	ZEB1 promotes tumorigenicity, invasion and chemoresistance against temozolomide	Siebzehnrubl et al., 2013 [58]
Glioma/GBM	TWIST1 promotes early glial tumorigenesis and subsequent malignant progression	Elias al., 2005 [59]; Mikheeva et al., 2010 [60]
Sarcoma	SNAIL expression provides tumorigenic capabilities to fibroblastic cells, whereas SNAIL depletion decreases sarcoma growth in a mouse model	Alba-Castellón et al., 2014 [61]
Sarcoma	Overexpression of ZEB1 relates to metastasis and invasion in osteosarcoma	Shen et al., 2012 [62]

*Abbreviations*: *DLBCL*, diffuse large B-cell lymphoma; *EMT*, epithelial-mesenchymal transition; *MM*, multiple myeloma; *NANOG*, NANOG homeobox; *NSCLC*, non small-cell lung carcinoma; *SNAIL*, Snail family transcriptional repressor 1; *SOX2*, SRY-box 2; *SLUG*, Snail family transcriptional repressor 2; *TGF-β*, transforming growth factor beta; *TWIST = TWIST1*, Twist family bHLH transcription factor 1; *ZEB1*, zinc finger E-box binding homeobox 1; *ZEB2*, zinc finger E-box binding homeobox 2

MITF plays an ambiguous but central role in melanoma (Fig. 1). On the one hand, MITF functions as proto-oncogene and plays a key role in cell cycle, cell survival and autophagy, DNA damage repair and metabolism [72, 73]. On the other hand, MITF is also a pigmentation and differentiation inducer, which explains the observation that melanoma stem cells and melanoblasts are amelanotic [74]. As a result, the MITF^high^ state yields in higher proliferative activity and a higher grade of differentiation with less invasive capacity. Conversely, melanoma cells exhibiting a MITF^low^ state are less proliferative but highly invasive and less differentiated [65, 75]. In this perspective, the potential back and forth flipping of single melanoma cells between MITF^high^ and MITF^low^ states characterizes cell plasticity [68, 76]. The ambiguous roles of EMT-TFs are further illustrated by the oncogenic effects of ZEB1 and TWIST1 versus the seemingly oncosuppressive effects of ZEB2 and SLUG, with MITF being their downstream target [67, 68]. Furthermore ZEB2 and SLUG are expressed in melanoblasts but also in melanocytes and benign nevi [67, 77]. Surprisingly SLUG is required for metastasis as metastasis is impeded by suppression of SLUG [78]. Similarly, the EMT-TF ZEB2 promotes the growth from micro- to macrometastasis by activating a proliferative transcriptional program, at the expense of invasiveness [79]. This paradox is explained by the observed phenotype switching from an invasive ZEB1/TWIST^high^MITF^low^ state towards an “anti-invasive” but proliferative ZEB2/SLUG^high^MITF^high^ state that allows macrometastatic outgrowth, similar as MET in carcinomas [26, 67, 78] (Fig. 1). This further supports the role of EMT/MET balancing (i.e., phenotype switching) in metastatic melanoma and underlines that EMT-TFs exert their oncobiologic roles in time and context-dependent manners that are somewhat counterintuitive [28]. For example, invasiveness is needed at the primary tumor of cutaneous melanoma, but to a much lesser degree at distant sites to elicit malignant behavior. Conversely, in order to elicit the ultimate malignant behavior—namely death—proliferation is more important at secondary metastatic sites.

The transcription factor BRN2 is another important central regulator in melanocytic development and melanomagenesis [80] (Fig. 1). BRN2 is a key player in phenotype switching as it drives transition into a dedifferentiated and slow-cycling but highly invasive state by transcriptionally repressing MITF and vice versa [81]. The BRN2 downstream transcription factor NFIB strongly promotes global chromatin accessibility *via* the histone modulator EZH2 and is in the same way directly inversely related to MITF [82]. It is therefore not surprising that EZH2 and NFIB overexpression is associated with invasion and adverse prognosis in melanoma, as EZH2 silences multiple tumor suppressor genes [82, 83]. Conversely, biallelic loss of the tumor suppressor *CDKN2A* (often lost in melanoma) dramatically increases BRN2 expression [84]. In accordance with the phenotype switching model, the BRN2/NFIB/EZH2-axis increases invasion but decreases proliferation [82]. This epigenetic mechanism is persuasive as it is dictated by microenvironmental cues and has the potential of reversibility that defines transdifferentiation, in a background of already fixed genetic driver mutations [6].

Again, reversion of the switch towards the “anti-invasive” but proliferative BRN2^low^MITF^high^ state allows a MET-like transition and metastatic outgrowth [80]. Yet, a biphenotypical switch is oversimplistic, as intermediate states also exist and contribute to intratumoral heterogeneity [85]. Based on relative expression of transcription factors like MITF and SOX10, six interconvertible distinct states were discovered recently: undifferentiated, NC stem cell (NCSC), intermediate, starved melanoma cell (SMC), melanocytic-like and the hyperdifferentiated/pigmented state [86] (Fig. 1). The six states were discovered by combined multiplex immunohistochemistry and single cell RNA sequencing and some of these indeed are invasive (undifferentiated and NCSC states), proliferative (melanocytic state) or both (intermediate and SMC states). The hyperdifferentiated state, a specifically drug-induced state, is an exception to the conventional biphenotypic switch as it does not retain proliferative nor invasive properties. Hyperdifferentiation following drug exposure is a known phenomenon in other tumors, e.g., in embryonal rhabdomyosarcoma where therapy effect induces selection of differentiated rhabdomyoblasts [87]. Alternatively, in a recent study it has been proposed that it might be more appropriate to view melanoma cells in a continuous spectrum of transcriptional states rather than distinct artificial categories [27] (Fig. 1).

## Tumor microenvironment remodeling and adhesion molecule alterations are involved in melanoma cell fate, motility, and migration

**Fig. 2:**
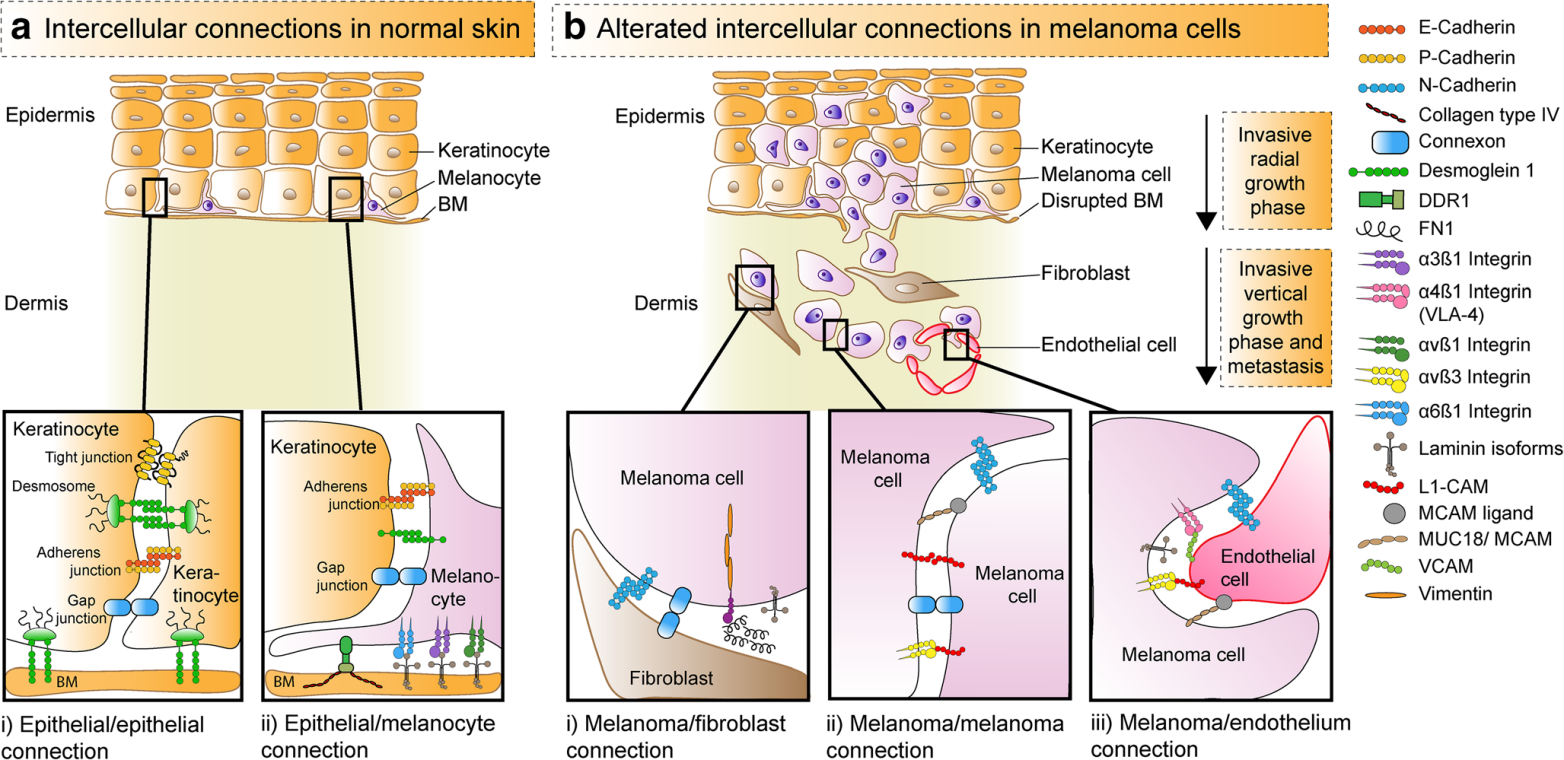
Cell-cell and cell-matrix adhesion of keratinocytes and melanocytes in normal skin versus melanoma. A) Intercellular contacts in epidermis with Ai) epithelial-epithelial and epithelial-basal membrane connections of keratinocytes (orange) and Aii) contacts of epidermal melanocytes (purple) with keratinocytes and basal membrane. Normal epidermal melanocytes interact with adjacent keratinocytes through E-cadherin, desmoglein 1, and gap junctions, which are formed by two connexons. B) Alterated adhesion pathways in melanoma with gain of motility during invasion. The first step in melanoma development is loss of connections between melanocytes with keratinocytes and basal membrane. Melanoma cells escape keratinocyte control and instead interact with Bi) fibroblasts (brown), Bii) other melanoma cells (purple), or Biii) endothelial cells (red), mainly during vertical growth phase that elicits the metastatic potential. Desmoglein 1 and other connections are disrupted while new adhesive and communication properties are conferred. Melanoma cells can express FN1 and the intermediate filament vimentin. They interact with fibroblasts Bi) through N-cadherin, FN1, gap junctions, and with other melanoma cells Bii) through αvβ3-integrin, L1-CAM, MUC18/MCAM, L1-CAM, gap junctions and N-cadherin. Transendothelial migration is mediated by adhesion of melanoma cells with endothelial cells Biii) through N-cadherin, MUC18/MCAM, MCAM ligand, α4β1-integrin, VCAM, αvβ3-integrin, and L1-CAM. Adopted from [88–91]. Abbreviations: BM, basement membrane; BRN2 = POU3F2, POU domain, class 3, transcription factor 2; CAF, cancer-associated fibroblast, FN1, fibronectin 1; L1-CAM, L1-cell adhesion molecule; MCAM, melanocyte cell adhesion molecule; OXPHOS, oxidative phosphorylation; SNAIL, Snail family transcriptional repressor 1; SLUG: Snail family transcriptional repressor 2; TWIST1 = TWIST, Twist family bHLH transcription factor 1; VCAM, vascular cell adhesion molecule; ZEB1, zinc finger E-box binding homeobox 1; ZEB2, zinc finger E-box binding homeobox 2

Differentiated melanocytes adhere to basal keratinocytes and the basal lamina *via* cell-cell and cell-matrix adhesion molecules like E-cadherin [88] (Fig. 2a). In contrast, transformed melanocytes undergo a cascade of changes that downregulate specific adhesion receptors and upregulate novel receptors, not found on melanocytes under normal conditions [88, 89]. Furthermore novel interactions between melanoma-melanoma cells, melanoma-fibroblast cells and melanoma-endothelial cells provide a gain in motility and migration, but also determine melanoma cell fate (Fig. 2b). Important interactions in melanoma are mediated by different laminin and integrin isoforms as well as several chemokine receptors [90, 92–95]. Integrins are a class of cell adhesion molecules that enable adhesion to other cells or the extracellular matrix [11, 89]. They are heterodimers composed of non-covalently linked α- and β-subunits. Some of these novel interactions ultimately predict organ specific tropism [93, 96]. Melanoma cells themselves can express melanocyte adhesion molecule (MCAM)/MUC18, L1-CAM, α4β1-integrin and αvβ3-integrin that promote transendothelial migration. Consequently, the association with metastatic disease is not surprising [97–99]. The onset of expression of MCAM/MUC18 and especially the β3-integrin subunit of αvβ3-integrin predicts progression from radial to vertical growth phase which elicits the metastatic potential in melanoma [89, 99]. In fact, the Breslow thickness remains the most significant prognostic stratifier of malignant melanoma today. At the same time, αvβ3-integrin is involved in extracellular matrix (ECM) degradation and immune evasion through programmed death ligand 1 (PD-L1) expression regulation [100, 101]. The switch of these adhesion molecules is once again mediated by EMT-TFs [69].

As already stated the five key steps of the metastatic cascade include invasion, intravasation, circulation, extravasation, and colonization at secondary sites [6]. Of these, it is known that metastatic colonization is the farmost inefficient step. Additionally, single disseminating cells are less successful than clusters (implying some sort of cohesion) [102]. Cohesion between melanoma cells is mediated by N-cadherin, αvβ3-integrin, L1-CAM, AL-CAM and MCAM/MUC18 that are not expressed on melanocytes [10, 88, 89, 98] (Fig. 2b).

Recent research has shown that CAFs that also express αvβ3-integrin can promote invasion through integrin-β3-dependent fibronectin assembly [103]. The various cells that shape the TME like CAFs, endothelial cells, macrophages and other leukocytes secrete cytokines and growth factors (TGF-β, IFN-y, TNF-α, VEGF, HGF, and others) that enable tumoral transformation in melanoma cells [12, 104–106]. The ECM remodeling molecule fibronectin-1 (FN1) is increasingly expressed not only on melanoma cells compared to benign nevi but also on metastatic versus primary melanoma [107, 108]. Furthermore, melanoma cells with FN1 expression are strongly associated with a pro-survival MITF^low^ state, upregulation of ZEB1 and hypoxia [109, 110]. Striking similarities are found in glypican-6, a heparan sulfate proteoglycan. Glypican-6 is recently proposed as a new putative biomarker of progression in melanoma [111]. Just as FN1, glypican-6 is upregulated in melanoma cells versus melanocytes and in metastatic versus primary melanoma [111]. Similarly, glypican-6 expression exhibited highest correlation with ZEB1 and is regulated by Hypoxia-inducible factor alpha (HIF1α)-signaling [111]. Hypoxia and acidity trigger invasion and dedifferentiation by HIF1α-mediated downregulation of MITF and consequent upregulation of ZEB1, SNAIL and the matrix metalloproteinases MMP2 and MMP9 [106, 112, 113]. As such, the dedifferentiation is in line with the observation that hypoxic melanoma cells are more often amelanotic [106]. In summary, the different conditions and components of the TME play principal roles in tumor progression in melanoma, but knowledge remains incomplete.

### The tumor microenvironment of Spitzoid melanocytic neoplasms might explain their unique behavior

Tissue stiffness and physical forces stemming from the ECM have an important impact on gene regulation and melanoma cell fate [114, 115]. Melanoma patients with increasing age have an adverse prognosis but paradoxically present less frequently with lymph node metastasis compared to younger patients [116]. This is in part due to age-related ECM remodeling that results in impaired lymphatic vasculature. This in turn might favor spread *via* the hematogenous route [117]. Notably, Spitzoid melanocytic neoplasms display this paradox to an even greater extent as they typically occur in younger patients and often present with locoregional disease but only very seldom with visceral metastases [118, 119]. Spitzoid neoplasms form a spectrum from strictly benign Spitz nevi to (exceptionally rare) malignant Spitz tumors [120]. Patients diagnosed with the intermediate category (atypical Spitz tumor) have an excellent prognosis, as visceral metastases are exceptionally rare. This is in line with the 5-year overall survival of 99% [119]. Remarkably, this stands in great contrast with the high rate (39%) of sentinel node positivity [119]. The mechanism behind this paradoxical behavior is unknown. Interestingly, Spitzoid neoplasms share a peculiar hypervascular TME, reminiscent of wound healing. The Spitzoid TME is therefore more than an epiphenomenon, but rather an important behavioral modificator [121]. Future research thus might be key to understand this uncoupling of lymphatic and hematogenous dissemination. Some authors even state that lymph node metastases are a bystander effect and nothing more than dead ends [122, 123]. Decades ago, Blake Cady already hypothesized this metaphorically: “…lymph node metastases are the speedometers of the oncologic vehicle, not the engine. Indicators, not governors” (cited from [124]). Also, multiple meta-analyses of randomized controlled trials show no survival benefit of lymph node dissection compared to observation in melanoma [125, 126]. This not only raises serious questions about today’s patient management, but also about our—lack of—understanding tumor biology.

## Melanoma oncometabolism and metabolic symbiosis with the stromal neighborhood

**Fig. 3:**
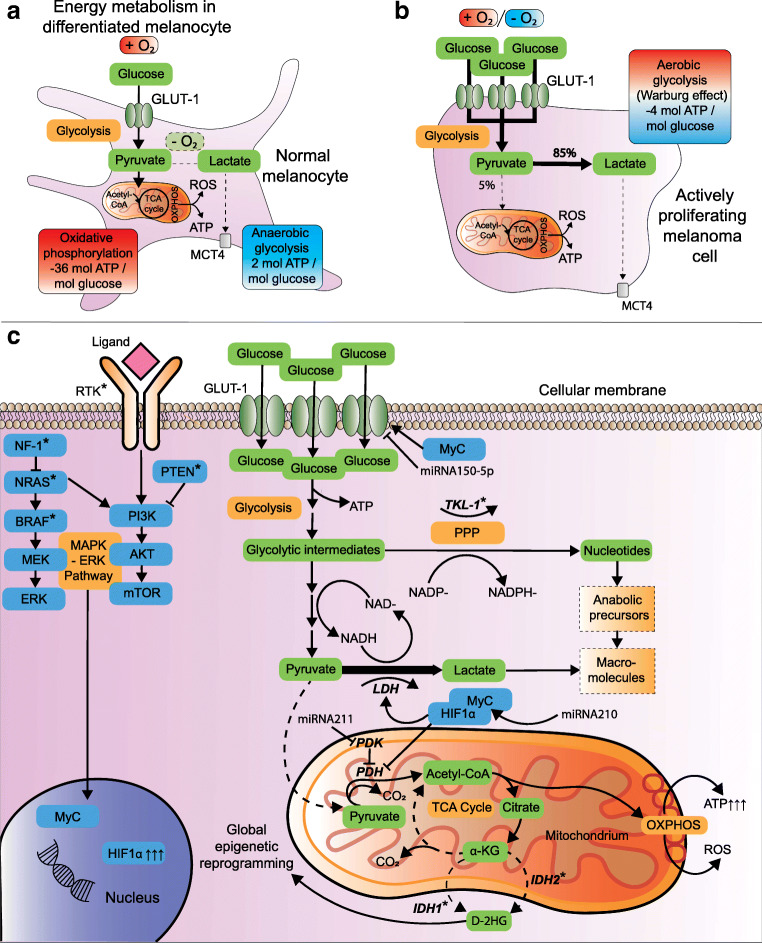
Metabolic reprogramming and the Warburg effect in melanoma. A) Metabolism in normal melanocyte with depiction of glycolysis, TCA cycle and OXPHOS. Glucose enters the cytoplasm *via* GLUT-1 and undergoes glycolysis to pyruvate. After entering the mitochondrium, the enzyme PDH converts pyruvate to Ac-CoA, which is metabolized and burnt in the TCA-cycle and during OXPHOS. Anaerobic glycolysis results in less efficient ATP generation along with lactate production during hypoxia. Lactate can leave the cytoplasm *via* MCTs. B) The Warburg effect refers to the metabolic switch in highly proliferating cells. Somewhat counterintuitive, these cells prefer less efficient ATP generating glycolysis as main metabolic pathway despite the presence of oxygen (i.e., aerobic glycolysis). C) Warburg effect is exploited by rapidly proliferating melanoma cells when nutrients are abundant. This is mediated by multiple mechanisms that result in increased glucose uptake, accelerated glycolytic flux and decreased mitochondrial respiration. Glycolytic intermediate metabolites can be recycled and synthesized into macromolecules for synthesis of DNA, lipids and cellular proteins that are needed for proliferation. Adopted from [128]. Abbreviations: Ac-CoA, acetyl coenzyme A; ATP, adenosine triphosphate; GLUT, glucose transporter; HIF1α, hypoxia-inducible factor alpha; MCT, monocarboxylate transporter; PDH, pyruvate dehydrogenase; PDK; PDH kinase; ROS, reactive oxygen species; TCA, tricarboxylic acid

Under physiological conditions, cellular energy in most cells is predominantly provided *via* oxidative phosphorylation (OXPHOS) in the mitochondria with generation of 36 mol adenosine triphosphate (ATP) per mol glucose [127] (Fig. 3a). An alternative, less efficient pathway is anaerobic glycolysis that generates lactate under hypoxic conditions with only 2 mol ATP per mol glucose (Fig. 3a). Metabolism of highly proliferative cells like proliferating lymphocytes or melanoma cells is dominated by aerobic glycolysis, i.e., preferred glycolysis with lactate production even under normoxic circumstances [129] (Fig. 3b). This is however more pronounced in malignancy and is known as the Warburg effect, already described in 1924 by Otto Warburg [130]. At first sight, the switch from OXPHOS to less ATP generating glycolysis appears paradoxical. However, aerobic glycolysis delivers ATP on top of recyclable intermediate metabolites for macromolecule biosynthesis [131] (Fig. 3c). Moreover, the generated lactate is more than a waste product as it is a valuable energy source, restores NAD+/NADH ratios and prevents conversion of cytosolic pyruvate to mitochondrial acetyl-coenzyme A (Ac-CoA) by pyruvate dehydrogenase (PDH). The Warburg effect pro-actively mitigates uncontrolled mitochondrial entry of metabolic intermediates that would generate excessive oxidative stress during OXPHOS [132, 133]. On the other hand, the inefficient ATP production during glycolysis compared to OXPHOS is (over)compensated in multiple ways. For example, HIF1α fuels Warburg effect by upregulation of glucose transporter-1 (GLUT-1), glycolytic enzymes, pyruvate dehydrogenase kinase (PDK) and lactate dehydrogenase A (LDHA) [129, 134, 135] (Fig. 3c). This results in accelerated glucose uptake, glycolytic flux, and decreased mitochondrial respiration [129].

Constitutive RAS/MAPK/ERK signaling—a hallmark of melanoma—stabilizes HIF1α [106, 136]. The effects of HIF1α in other cancers are well known and one of the avenues towards successful neo-angiogenesis, but also oncometabolism, EMT and metastasis. This is in part due to increased expression of EMT-TFs such as TWIST [137, 138] (see Section 1). Oncometabolism in melanoma is also controlled by micro-RNAs (miRs), which are small non-coding RNA sequences that regulate gene expression through mRNA target degradation or inhibition of mRNA translation. To date, miR-210 is one of the most prominent upregulated miRs in hypoxic melanoma cells [139]. Overexpression of miR-210 in melanoma induces bypass of hypoxia-induced cell cycle arrest together with a MYC-like transcriptional response [139]. MYC is a master transcription factor that boosts metabolism and proliferation in various ways.

On the other hand miR-150-5p is a tumor suppressive miR that dampens glucose uptake and glycolysis [140]. In parallel with the latter, miR-211 destabilizes HIF1α and is often downregulated in melanoma [141]. Importantly, an additional effect of miR-211 is inhibition of the isoenzyme pyruvate dehydrogenase kinase 4 (PDK4) [141]. This enzyme prevents mitochondrial respiration in favor of glycolysis *via* inhibition of PDH (Fig. 3c). Other PDK isoforms—PDK1 and PDK3—inhibit PDH as well, fueling Warburg effect in melanoma [133, 142]. Therefore, the tumor suppressive miR-211 is a central metabolic switch that attenuates Warburg effect. *Ergo*, when miR-211 and miR-150-5p are downregulated, glycolytic intermediates and lactate accumulate and are available for biomass incorporation. Interestingly, miR-211 also inhibits BRN2 [80]. This transcription factor induces phenotype switching in melanoma [80–82] (see Section 2). In this respect, miR-211 couples a metabolic switch with phenotype switching. The rapidly growing list of miRs in melanoma is further reviewed elsewhere [143]. Another essential element of Warburg effect in melanoma is reactivation of transketolase-like 1 (*TKL-1*). This gene is silenced under physiological conditions but is reactivated through promoter hypomethylation in melanoma [144]. The enzyme TKL-1 shuttles intermediate metabolites from (aerobic) glycolysis to the pentose-phosphate pathway (PPP). The latter not only generates NADPH, an essential cofactor for synthesis of various biomolecules like lipids, but also induces the oxygen-independent conversion of glucose to ribose-5P, an essential biomolecule for nucleic acid synthesis [144] (Fig. 3c).

The metastatic suppressor gene *KISS1* exerts its role metabolically as it enhances mitochondrial biogenesis and respiration and simultaneously decreases Warburg effect [145]. Initially Otto Warburg blamed defective mitochondrial respiration for enhanced aerobic glycolysis [146]. Nonetheless, recent research suggests a critical role of highly functioning mitochondria in metastatic disease and therapy resistance [147–149]. These observations are supported by an increase in mitochondrial mass, DNA content, and reactive oxygen species (ROS) production [150, 151]. In cell cultured melanoma cells that are depleted of mitochondrial DNA, formation of new tumors is only possible after obtaining mitochondrial DNA from host cells [152]. Additionally, retrograde signaling from mitochondria to the nucleus can induce posttranslational modifications together with modified transcriptional regulation [153]. Mutations in enzymes of the tricarboxylic acid (TCA) cycle lead to accumulation of oncometabolites that facilitate malignant transformation and metastasis [154, 155]. For instance, mutations of isocitrate dehydrogenase (*IDH*) are well known in certain malignancies and generate the oncometabolite D-2-hydroxyglutarate (D-2HG) [156, 157]. About 10% of melanomas harbor *IDH1* or *IDH2* mutations that contribute to transformation by global epigenetic reprogramming [131, 158–160].

**Fig. 4:**
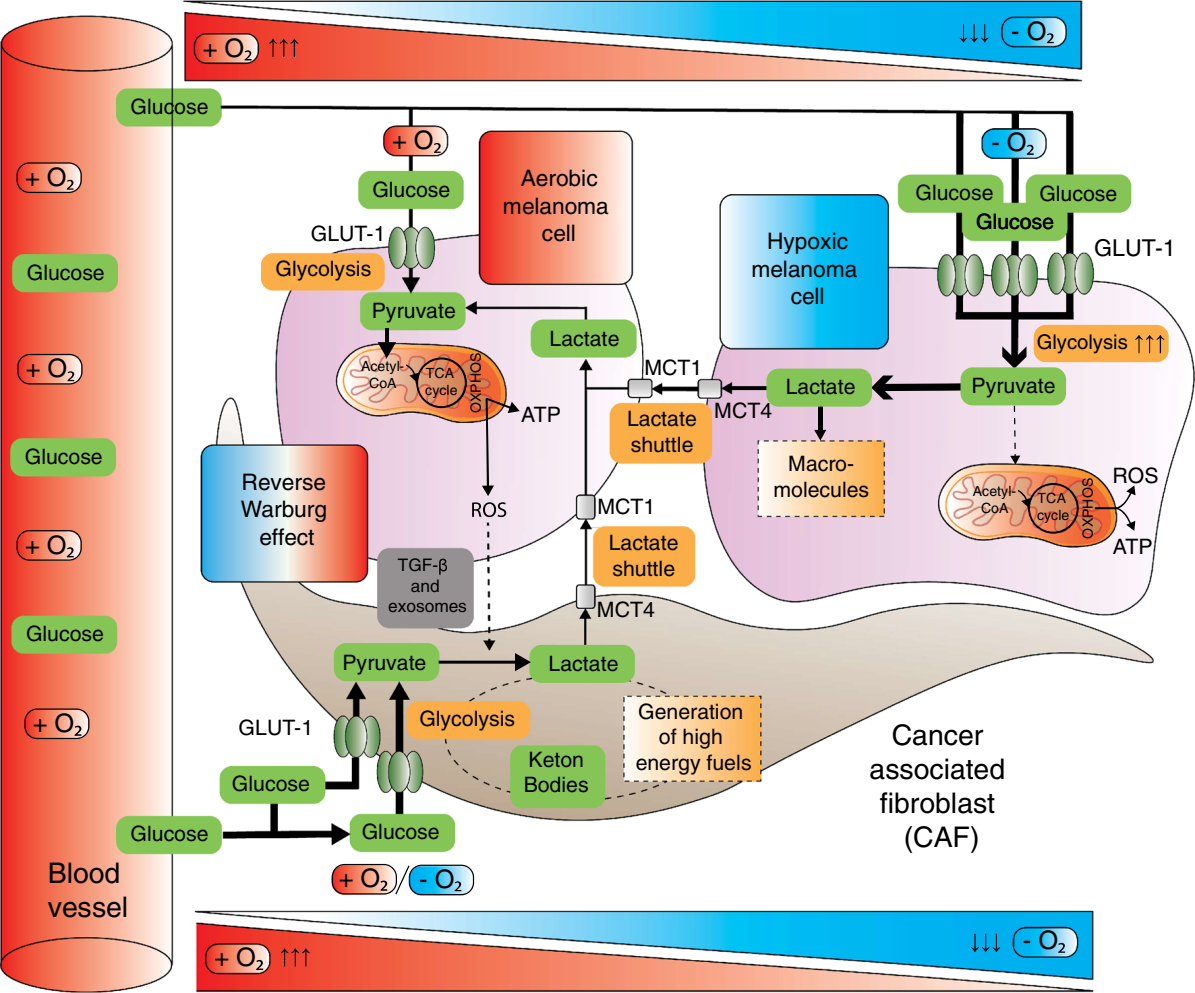
Metabolic symbiosis as a result of nutrient trade-off between different melanoma cell subsets and adjacent CAFs. Stromal symbiosis is the result of (unidirectional) nutrient trade-off by CAFs and melanoma cells (reverse Warburg effect). This is mediated by aerobic glycolysis in adjacent CAFs that produce metabolites like lactate, pyruvate and ketone bodies. These metabolites are shuttled through MCTs to sustain the anabolism of adjacent melanoma cells. An additional effect of the exploited lactate shuttling is the symbiosis between better and worse oxygenated melanoma cells. Hypoxic melanoma cells produce more lactate that is preferentially taken up and metabolized by better oxygenated melanoma cells. The latter in turn spare the glucose for hypoxic melanoma cells. Adopted from [15, 19]. Abbreviations: Ac-CoA, acetyl coenzyme A; ATP, adenosine triphosphate; CAF, cancer-associated fibroblast; GLUT, glucose transporter; HIF1α, hypoxia-inducible factor 1 alpha; MCT, monocarboxylate transporter; PDH, pyruvate dehydrogenase; PDK, PDH kinase; ROS, reactive oxygen species, TCA, tricarboxylic acid

Of note, the dominant metabolic phenotype does not have to be uniform spatiotemporally [14, 15, 161, 162]. Similar as in phenotype switching, melanomas can adapt their metabolism and oxygen use by shifting from one pathway to another or by acquiring intermediate metabolic states depending on microenvironmental alterations [13, 15, 128]. Slow-cycling melanoma populations exhibit elevated mitochondrial mass and OXPHOS [14, 148]. This hybrid metabolic signature contributes to tumor plasticity and provides multiple advantages [163]. First of all, melanoma cells in different microenvironments gain flexibility by balancing the maximal proliferative capacity when nutrients are abundant against the minimal required ATP production that allows survival under marginal conditions. This is mediated through Warburg metabolism in rapidly proliferating cells versus OXPHOS in starving or dormant melanoma cells [14, 129, 164]. This is known as the proliferation/survival trade-off [23]. Secondly, the hybrid metabolic phenotype balances ROS at steady level. This balance avoids an excessively high toxic ROS production but still generates enough ROS to create a mutation prone milieu. Finally, the hybrid state increases phenotypic tumor heterogeneity [149, 165, 166]. For example, functional differences of the lactate transporter monocarboxylate transporter-1 (MCT1) enhance metastatic potential by contributing to metabolic heterogeneity in melanoma [15]. In this manner, subclones benefit from increased tolerability of oxidative stress that peaks during dissemination [106, 132]. Concurrently, phenotypic heterogeneity facilitates metabolic symbiosis between hypoxic and aerobic melanoma cells [15, 164] (Fig. 4). Aerobic tumor cells prefer lactate over glucose as an energy source. As a result, glucose is spared for hypoxic tumor cells, which in turn produce lactate on a larger scale [18, 132].

Importantly, cooperation of melanoma cells with the CAFs that shape the TME is key in metabolic plasticity [167]. Metabolic symbiosis and reprogramming is in fact not limited to cancer cells: CAFs are exploited by cancer cells to undergo aerobic glycolysis themselves in a host-parasite relationship. This synergistic effect is also known as the reverse Warburg effect [19] (Fig. 4). TGF-β, ROS and exosomes stimulate aerobic glycolysis in CAFs [20, 168, 169]. They thereby amplify glucose uptake and generate lactate, pyruvate and other energetic metabolites that can be unidirectionally transferred to cancer cells. Both the classic Warburg affect and the reverse Warburg effect likely co-occur [19, 170–172].

## **Dormancy**—**the state of cellular deep sleep impacting the onset of metastatic outgrowth and therapy response**

The relation and difference between cancer stem cells (CSCs), quiescent and dormant cells is not always clear as these terms are often used interchangeably in the context of malignancy. Quiescence (G_0_) is—in contrast to senescence—a state of reversible cell cycle arrest [173]. During tumor cell quiescence, cell cycle is paused in order to repair damage or to sustain elevated stressors like nutrient scarcity or therapy effects [174]. Dormant tumor cells might be compared to a special kind of stem cells in a quiescent state [173]. These cells can remain silent for weeks, years or decades and are ultimately the reason for delayed metastatic disease [175, 176]. A relatively small but continuously growing number of metastatic suppressor genes is arising. These genes repress metastasis without effect on the primary tumor. In melanoma the most important genes include *GAS1*, *BRMS1*, *nm23*, *KISS1*, *KAI1* (CD82), *SSeCKS*, *SMAD7*, and *Gelsolin* [145, 177–183]. As one can expect, some of these genes (e.g., *Gelsolin*, *SMAD7*, *KISS1*, *nm23)* are implied in dormancy [145, 182, 184]. A vital question is what awakes dormant tumor cells. On a molecular level, the stress-activated protein kinase (SAPK) p38 induces dormancy and is anticorrelated with ERK [185]. As a result, a low ERK/p38 ratio induces dormancy in most tumors [185]. In contrast to carcinomas, a high p38 activity does not have to lead to dormancy or decreased ERK signaling in melanomas per se [185, 186]. Typical driver mutations in melanoma such as *BRAF*, *NRAS*, and *KIT* result in constitutive MEK-ERK-signaling. The underlying molecular signaling cascades involving dormancy are beyond the scope of this review and are discussed elsewhere [22, 187–189].

Three non-mutually exclusive dormancy models have been proposed [173, 187] (Fig. 4). There is (i) cellular dormancy, in which single cells enter quiescence, meaning that there is no significant proliferation (low/absent Ki-67 expression). The two other model components are processes where the fate of a tumor mass is balanced between cell proliferation or cell death, caused by (ii) insufficient vascularization (angiogenic dormancy) and/or (iii) immune-mediated melanoma cell lysis (immunogenic dormancy). Conversely, dormancy escape due to intrinsic or extrinsic signals is featured by proliferation, neoangiogenesis, and/or immune evasion.

The observation that (immune compromised) organ recipients develop melanoma if donors had a history of melanoma—but were disease “free” for years at time of transplantation—strongly underlines the role of immunogenic dormancy in melanoma [190, 191]. Rather than NK or CD8+ cytotoxic T-cell mediated tumor cell killing, an immune mediated cytostatic effect likely trumps cytotoxic cell killing in immunogenic dormancy [192]. Additionally, helper T-cells, cytotoxic T-cells and NK cells secrete IFN-y and TNF-α that are antiproliferative and drive immunogenic dormancy [193]. Moreover helper T-cells secrete anti-angiogenic chemokines and prevent αvβ3-integrin expression on melanoma cells [91, 194]. Apart from the immunogenic nature of melanomas, the composition, distribution, density and activation status of tumor infiltrating leukocytes defines the immunoreactivity of melanocytic lesions. Therefore tumor infiltrating leukocytes are prognostically relevant, modify behavior and are exploitable in immunotherapy [195, 196].

**Fig. 5:**
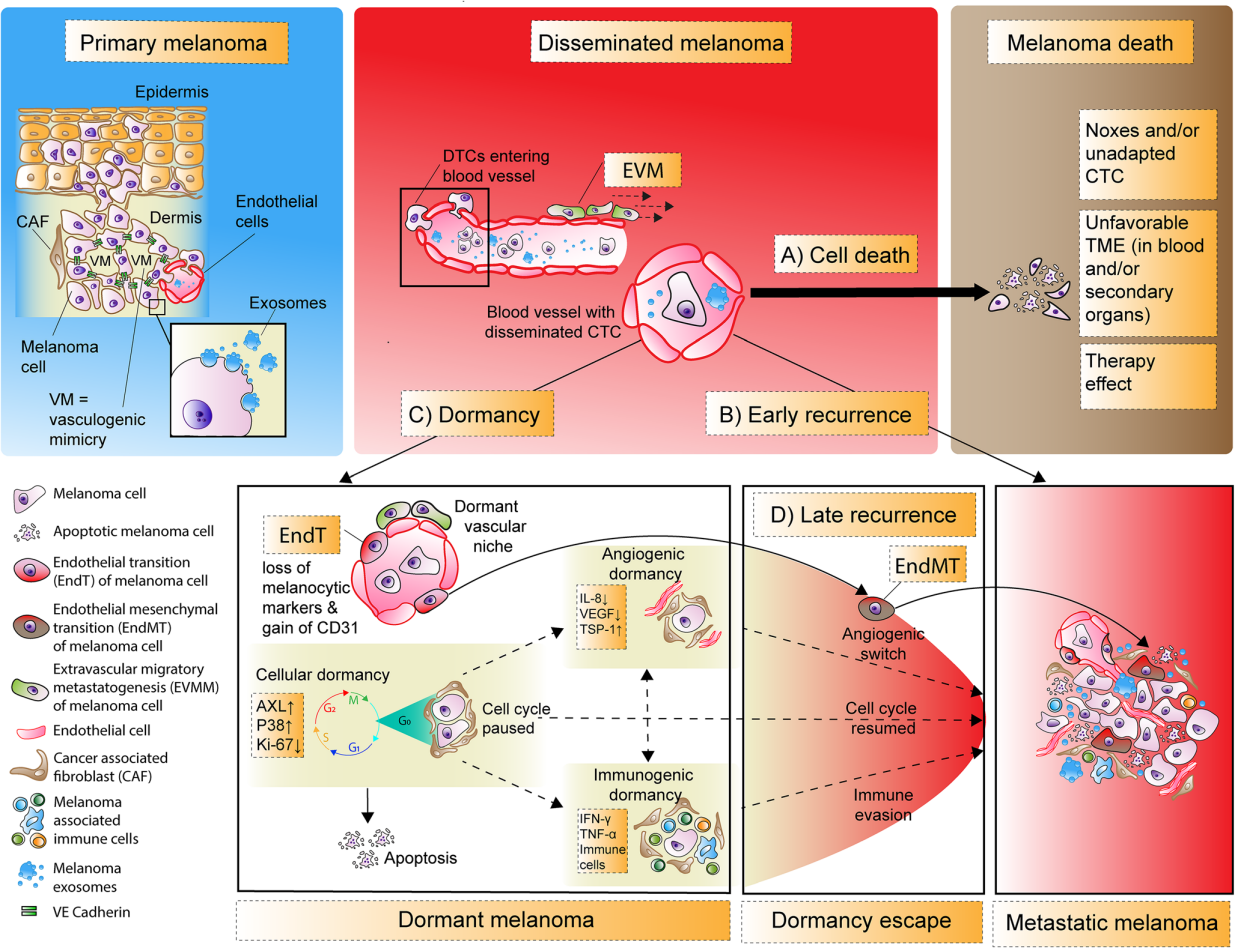
The role of dormancy in melanoma. The majority of disseminated melanoma cells die (A); however, a fraction possesses the potential to adapt to various new environments, followed either by early recurrence (B) with metastasis or induction of dormancy (C), which can yield in late recurrence (D) of metastatic disease. At the primary tumor aggressive subclones can dedifferentiate to mimic vascular channels, delined by melanoma cells that express VE-cadherin in a process named vasculogenic mimicry. After dissemination, invasive melanoma cells can migrate intra- or perivascularly and as single cells or cell clusters. The vascular niche comprises the non-mutually exclusive extravascular and intravascular niche. The former involves the migration of melanoma cells *via* the abluminal side of pre-existing vascular structures *via* extravascular migration (EVM). Eventually this leads to metastatic outgrowth at secondary sites through extravascular migratory metastasis (EVMM). Dormancy can be subdivided in cellular dormancy and tumor mass dormancy due to angiostasis (angiogenic dormancy) or immuno surveillance (immunogenic dormancy). Dormant melanoma cells can reside for years at their dormant niches and potentially transdifferentiate to endothelial cells by endothelial transition (EndT), where melanocytic markers are lost and the endothelial marker CD31 is gained. Intrinsic and/or extrinsic factors ultimately induce an outbreak from dormancy, thereby promoting a clinically visible and/or symptomatic metastasic state of the disease. The essential prerequisite for metastasis is that the surrounding new TME allows the adapted or adaptive melanoma cells to survive. Shedded exosomes help to create a cancer-friendly secondary TME which ultimately leads to organotropism. Escape from dormancy is the result of immune evasion, angiogenic switch and/or EndMT and will ultimately lead to a metastatic state of the disease. Finally, in the (macro)metastatic state, metabolic alterations of melanoma cells and their TME such as the (reverse) Warburg effect become significant. Abbreviations: CAF, cancer-associated fibroblast; CTC, circulation tumor cells; ECM, extracellular matrix; DTC, disseminated tumor cells; EVM, extravascular migration; EVMM, extravascular migratory metastasis; EndT, endothelial transition; EndMT, endothelial mesenchymal transition, VE-cadherin, vascular-endothelial cadherin

Escape from angiogenic dormancy also occurs when the balance of pro- and anti-angiogenic factors is disrupted, allowing nutrition and oxygen levels to sustain not merely survival but also proliferation. TME stressors like ROS and hypoxia promote an angiogenic switch through stromal production of VEGF, IL-8 and FGF whereas thrombospondin-1 (TSP-1) keeps melanoma cells in a dormant state [197, 198]. Research has shown that the angiostatic factor TSP-1 prevents outgrowth of dormant micrometastases in human melanoma xenografts [199, 200]. Additionally, real-time imaging of human melanoma xenografts in murine brains showed peri/extravascular migration (EVM) of human melanoma disseminated tumor cells (DTCs). This occurred *via* pre-existing vascular networks, in contrast to *de novo* vessel formation in neoangiogenesis [201]. In this process of vessel co-option, melanoma cells reside preferentially in a perivascular niche where nutrients and oxygen are abundant. Furthermore, a preferential reservoir and niche formation of dormant melanoma cells is also apparent in lung and bone marrow perivascular regions as well as the atrophied thymus [188, 202]. At the same time, recent work provides proof for dormant intravascular niches at (pre)metastatic sites in melanoma [203]. Here, *in vivo* lineage tracing showed that subpopulations of melanoma DTCs can lose melanocytic markers and acquire the endothelial marker CD31. These subpopulations can undergo endothelial transition (EndT) and enter dormancy. Interestingly, these transdifferentiated melanoma cells are disguised and inert to immune surveillance, but can reawaken to escape dormancy by endothelial mesenchymal transition (EndMT) [203] (Fig. 5).

In contrast to vascular co-option and endothelial transdifferentiation, vasculogenic mimicry occurs within the cancerous tissue and has gained interest due to its potential role as a therapeutic target [204]. Vasculogenic mimicry is defined as the neoformation of fluid-conducting vascular-like channels. This alternative mechanism of tumor perfusion was first described in melanoma cell lines [205]. The vascular networks in vasculogenic mimicry are not lined by traditional endothelial cells but instead are formed by the dediffererentiated aggressive melanoma subpopulations [205, 206]. Interestingly, these melanoma subpopulations share a plastic partial transendothelial phenotype as they gain the endothelial adhesion molecule vascular endothelial (VE)-cadherin [206].

DTCs are measurable in the blood as circulating tumor cells (CTCs) or cell free circulating tumor DNA (ctDNA). These are already measurable very early in tumorigenesis, probably even before clinical detection of the primary tumor. This stands in stark contrast with late occurring metastatic disease, sometimes observed in melanoma [207]. However, the blood compartment is a notoriously stressful environment for CTCs, considering that < 0.1 % of CTCs in melanoma animal models metastasize [21]. As invasive melanoma cells enter the blood, phenotype switching leads to a gain in mesenchymal traits [16]. Their new liquid microenvironment lacks the supportive stroma of the primary tumor including metabolic fueling by the reverse Warburg effect. If melanoma CTCs enter dormancy, this is accompanied by a virtually non-proliferative MITF^low^ phenotype [12, 75] (see Section 2). None to only a small fraction of circulating melanoma cells retain proliferative capacity resulting in early metastatic disease. Notably AXL upregulation is associated with MITF^low^ state in *NRAS*- and *BRAF*-mutated melanomas leading to increased survival, therapy resistance and dormancy [208, 209]. Consequently, this emphasizes the connection between dormancy and melanoma cell plasticity.

Apart from the tumor specific dormant niches, metastatic suppressor genes and the three non-mutually exclusive dormancy subtypes, an accumulating amount of data suggests that secondary metastatic sites are not passive receivers of DTCs. In the next section we will discuss the role of primary melanoma-derived exosomes that induce preconditioning of secondary sites to foster colonization.

## Melanoma-derived extracellular vesicles and premetastatic niche formation

Pioneering findings in melanoma research illustrated the relevance of premetastatic niche formation in metastatic disease [25]. Premetastatic niches result from the distant effects of tumor secreted soluble factors and extracellular vesicles of which *exosomes* are the most relevant ones [210]. Exosomes measure 30–150 nm and can be horizontally transferred to recipient cells. In melanoma these extracellular vesicles contain bioactive molecules consisting of metabolites, (glyco)proteins, and genetic material including DNA as well as coding and non-coding RNAs [25, 211, 212]. Exosomes secreted by melanoma cells recruit and consequently educate non-resident cells, such as bone marrow-derived cells (BMDCs) to the premetastatic niches at distant organs [25, 210]. These BMDCs secrete soluble factors like MMP-9 that reshape the premetastatic niche and facilitate colonization of CTCs [213, 214]. Eventually this cascade leads to vascular leakage and inflammation at the premetastatic niche. Nevertheless, not all premetastatic niches are immediately compatible with metastatic growth. Some rather promote dormancy of colonizing DTCs and are so called “sleepy niches” [210].

Stromal cells at distant sites that have taken up exosomes become metabolically reprogrammed [20]. Their metabolism will shift towards aerobic glycolysis, comparable with the metabolic rewiring of CAFs at the primary melanoma and the reverse Warburg effect [20]. Of importance, fibroblasts as well as endothelial cells at the premetastatic niche remodel the ECM by fibronectin deposition [213]. The hallmark study of Kaplan et al. served first proof for a lung premetastatic niche formation in melanoma, which is made possible by BMDCs that—just as melanomas—express VLA-4 (α4β1-integrin), a known fibronectin ligand [210, 215]. Moreover, melanoma-derived exosomes express integrins (α4β1-integrin and αvβ3-integrin) as well. These are ligands of fibronectin, VCAM, and L1-CAM among others [25, 88, 96, 103] (see Section 3). Melanoma-derived exosomes also contain the receptor tyrosine kinases (RTKs) ALK and MET which are horizontally transferred to other target cells like BMDCs [25, 216]. The effects mediated by exosomes are not only organotropic, enabling specific visceral metastasis, but also drive early lymph node metastasis [24, 215, 217]. Last but not least exosomes can also induce phenotype switching in melanoma [218]. In summary, exosomes play a crucial role in melanoma and metastasis formation by induction of phenotype switching [218, 219] (Section 2), metabolic rewiring [20] (Section 4), dormancy [210] (Section 5) and TME remodeling including cell adhesion molecule alterations at the primary tumor (Section 3) as well as at secondary sites [20, 96, 213]. The latter is facilitated by premetastatic niche formation as discussed in this section (Section 6). This further consolidates the concept of obligate concurrence of the abovementioned sections of this review.

## Summary and future research objectives

This review dissected and at the same time integrated the cardinal driving forces of melanomagenesis and metastasis formation. First, this includes phenotype switching that gives rise to various differentiation states that enable tumorigenesis, invasion, survival in the circulation and metastatic outgrowth. Secondly, we discussed melanoma cellular adhesion pathways that provide motility and metastatic potential. Thirdly, we discussed the hybrid metabolic signature that comprises highly proliferative melanoma cells exploiting the Warburg effect, balanced with slow-cycling melanoma cells that reutilize OXPHOS. Apart from metabolic reprogramming in melanoma cells, there is synergy with the stroma as well as interphenotypic nutrient trade-off. Finally, we discussed dormancy and its reverted state—dormancy escape—in conjunction with premetastatic niche formation by melanoma cell shedded exosomes.

The interconnection of the aforementioned biological concepts is illustrated by the fact that phenotype switching also leads to a metabolic switch. For instance hypoxia and nutrient scarcity are less compatible with a proliferative MITF^high^ state, thereby skewing cellular machinery towards a metabolic restrictive but survival compatible MITF^low^ state. Another example is the already mentioned metastatic suppressor *KISS1* that mitigates Warburg metabolism in melanoma [145]. Biallelic *KISS1* loss also leads to awakening of dormant tumor cells and by consequence couples metabolic rewiring with dormancy (escape) and metastatic outgrowth [184]. Cohesion between melanoma cells is mediated by various surface molecules. At first sight this seems counterintuitive since the exact opposite—discohesion—is essential for invasion. Nevertheless cohesion between melanoma cells enables better survival in the blood and at distant organs, but also enables intercellular communication and metabolic symbiosis [15, 16, 128]. Hence, this demonstrates the earlier mentioned time and context-dependent aspects during melanomagenesis.

As opposed to the exponentially growing knowledge of tumor biology, durable curative therapies are dramatically lagging behind in the treatment of metastatic melanoma. The interplay between the tumor microenvironment and (epi)genetic mutations enables phenotype switching that drives cellular plasticity and intratumor heterogeneity. In melanoma this appears to occur in great extent and hinders long lasting clinical response [220]. On the one hand, the increased intratumor heterogeneity increases the probability of rare melanoma subclones that may be endowed with an intrinsic ability to metastasize or exhibit therapy resistance. On the other hand and probably more important, the phenotype switching model explains the unique adaptive capacity of melanoma cells that may be able to overcome environmental stressors like current therapies. Therefore, future research should take into account that intratumor heterogeneity and cellular plasticity represent the major barriers in targeting metastatic melanoma effectively, realizing that some treatments might induce phenotype switching towards treatment-resistant subpopulations.

Solely focusing on killing metastatic cells with antimitotic agents often leads to therapy-resistant MRD, in part because proliferating cancer cells may enter a dormant state as a self-defense mechanism [221]. Besides, cancer cells that are already dormant have higher tolerance to conventional pharmaceutical agents. As circulating dormant tumor cells are responsible for eventual metastatic outgrowth, novel therapies might better focus on either eliminating these cells or on sustainability of dormancy to inhibit dormancy escape. For example, treatment with alkylating drugs like cisplatin and BRAF/MEK inhibition uniformly leads to enrichment of slow-cycling melanoma cells that switch metabolically to OXPHOS [148]. Therefore combination therapies that also tackle mitochondrial respiration show promising results [148, 151]. Recently, co-occurrence of four different drug-tolerant mitotically inactive transcriptional states were discovered by bulk RNA sequencing after BRAF/MEK inhibition [5]. These include the SMC, NCSC, invasive, and hyperdifferentiated/pigmented melanoma cell states (Fig. 1). Further drug exposure leads to selection of the NCSC state that is responsible for relapse. The NCSC state is largely driven by the nuclear receptor RXRG and therefore RXR-antagonists might be of valid use to eliminate MRD in melanoma [5].

As already described before, the NC gives rise to transient migratory melanoblasts. In the vertebrate embryo, melanoblasts can use external surfaces of blood vessels as guidewires in an angiotropic fashion called extravascular migration (EVM) [222, 223] (Fig. 5). Recently, it has been shown that subsets of melanomas can spread by this mechanism, i.e., extravascular migratory metastasis (EVMM). EVMM can thus be viewed as a reversion towards the NC-related embryonic migratory phenotype [223, 224]. This idea is in line with transcriptome analysis of metastatic melanoma that shows striking similarities to melanoblasts [225]. Notably, the way of spread is not intravascular nor lymphatic and is by consequence an alternative mechanism of metastasis formation [223, 226]. Despite controversy, this mechanism is gaining interest and is also postulated in Spitzoid melanocytic neoplasms [227].

The close interplay and similarities between malignant tumors and embryologic development was already proposed by Rudolf Virchow in 1859 [228]. In melanoma, this is even more straightforward as the melanoblast emigrates through the vertebrate embryo and melanoma cells revive this migration program to elicit malignancy. Despite the multiple resemblances, perhaps the most striking difference between embryology and malignancy is the highly coordinated and deterministic integrity of embryological development. This stands in stark contrast with the highly inefficient and virtually stochastic manner of metastasis formation that takes away a life, instead of creating one.

## Conclusion

Metastasis formation is the main determinant of cancer therapy failure along with mortality. Despite decades of advanced research, it remains poorly understood. This is in part due to the co-occurrence of multiple distinct processes by which an integrative approach is overlooked. Put differently, when considering melanomagenesis, phenotype switching, metabolic reprogramming, stromal symbiosis as well as dormancy are not mutually exclusive. As a matter of fact the opposite is true and at the same time, the reason for metastatic success. The underlying reason is the high level of intratumor heterogeneity along with tumor cell plasticity that defines the highly dynamic potential to overcome exogenous stressors like current therapies. In this point of view, different strategies to tackle metastatic disease are urgently needed.

## Abbreviations

αSMA: ALPHA-smooth muscle actin
Ac-CoA: acetyl-coenzyme A
ATP: adenosine triphosphate
BM: basement membrane
BMDC: bone marrow-derived cell
BRN2: alias POU3F2, POU domain, class 3, transcription factor 2
CAF: cancer-associated fibroblast
CAM: cell adhesion molecule
CSC: cancer stem cell
CTC: circulating tumor cell
D-2HG: D-2-hydroxyglutarate
DLBCL: diffuse large B-cell lymphoma
DTC: disseminated tumor cell
ECM: extracellular matrix
EMT: epithelial-mesenchymal transition
EMT-TF: epithelial-mesenchymal transition transcription factors
EndMT: endothelial mesenchymal transition
EndT: endothelial transition
EVM: extravascular migration
EVMM: extravascular migratory metastasis
EZH2: enhancer of zeste homolog 2
FGF: fibroblast growth factor
FN-1: fibronectin-1
GLUT1: glucose transporter 1
HGF: hepatocyte growth factor
HIF1α: hypoxia-inducible factor 1 alpha
HMB45: human melanoma black 45
IDH: isocitrate dehydrogenase
IFNγ: interferon gamma
IL-6: interleukin 6
LDHA: lactate dehydrogenase A
MAPK: mitogen activated protein kinase
MCAM: melanocyte adhesion molecule, alias MUC18
MCT1: monocarboxyl transporter 1
MET: mesenchymal epithelial transition, not to be confused with the receptor tyrosine kinase MET
miRNA: micro-RNA
MITF: microphthalmia-associated transcription factor
MM: multiple myeloma
MMP: matrix metalloproteinase
MRD: minimal residual disease
NAD(P)H: nicotinamide adenine dinucleotide (phosphate)
NC: neural crest
NCSC: neural crest stem cell
NFIB: nuclear factor 1 B-type
NF-1: neurofibromin 1
NK: natural killer cell
NSCLC: non small-cell lung carcinoma
OXPHOS: oxidative phosphorylation
PDH: pyruvate dehydrogenase
PDK: pyruvate dehydrogenase kinase
PD-L1: programmed death ligand 1
PI3K: phosphoinositol 3 kinase
PPP: pentose-phosphate pathway
ROS: reactive oxygen species
RTK: receptor tyrosine kinase
RXRG: retinoid X receptor gamma
SAPK: stress-activated protein kinase
SLUG/SNAI2: Snail family transcriptional repressor 2 (alias symbols: SNAI2, SLUGH1, SNAIL2, WS2D, SLUGH)
SMAD: SMA = small body size and MAD = mothers against decapentaplegic
SMC: starved melanoma cell
SNAIL/SNAI1: Snail family transcriptional repressor 1 (alias symbols: SNA, SLUGH2, SNAH, SNAIL1, SNAIL)
SOX2: SRY-box 2
TCA: transcarboxylic acid cycle (= Krebs cycle)
TGF-β: transforming growth factor beta
TKL-1: transketolase-1
TME: tumor microenvironment
TNFα: tumor necrosis factor alpha
TSP-1: thrombospondin-1
TWIST1 = TWIST: Twist family bHLH transcription factor 1
TWIST2: Twist family bHLH transcription factor 2
VCAM: vascular cell adhesion molecule
VEGF: vascular endothelial growth factor
VE-cadherin: vascular-endothelial cadherin
ZEB1: zinc finger E-box binding homeobox 1
ZEB2: zinc finger E-box binding homeobox 2
